# Adverse Childhood Experiences and Diabetes Risk in Mississippi Adults

**DOI:** 10.7759/cureus.55875

**Published:** 2024-03-09

**Authors:** Zachary Boswell, Christopher Williams, Jamil Abdo, Roy Chedid, Danielle Fastring

**Affiliations:** 1 College of Osteopathic Medicine, William Carey University, Hattiesburg, USA

**Keywords:** type 2 diabetes mellitus (dm), diabetes in mississippi, adverse childhood experiences (aces), diabetes risk factors, ace categories, cumulative ace exposure

## Abstract

Despite Mississippi's high diabetes prevalence and the growing literature finding significant associations between adverse childhood experiences (ACEs) and diabetes, no research has examined the relationship between ACEs and diabetes risk in Mississippi adults. This study utilized data from the 2020 Behavioral Risk Factor Surveillance System (BRFSS) to determine if such a relationship existed. Data for Mississippi respondents were weighted to account for nonresponse bias and non-coverage errors.

Each respondent's total ACE exposure score was calculated based on the number of ACE categories experienced. Multivariate logistic regression was utilized to model the relationship between diabetes and ACE categories and diabetes and total ACE exposure scores. Variables that were significant at p<0.05 were retained in the final (best-fitting) models. All models were adjusted for sex, age, race, level of education, income, and body mass index (BMI). After adjusting for covariates, those experiencing physical abuse (adjusted odds ratio (AOR) 1.72, 95% CI 1.69; 1.75) or sexual abuse (AOR 1.56, 95% CI 1.53; 1.58) had the highest odds of ever being diagnosed with diabetes. Experiencing one ACE (AOR 1.02, 95% CI 1.01; 1.03) was associated with slightly higher odds of having diabetes, while experiencing seven ACE categories (AOR 2.20, 95% CI 2.10; 2.31) had the highest odds. Overall, this study shows a strong association between ACEs and a diagnosis of diabetes in the state of Mississippi. This relationship represents an important focus area for prevention efforts in legislation, public health campaigns, and universal screening procedures in primary care that may decrease the prevalence and burden of diabetes in Mississippi.

## Introduction

In 2018, the Centers for Disease Control and Prevention (CDC) reported that 12.9% of Mississippi residents aged 18-79 were diagnosed with diabetes, the second highest percentage in the United States [1]. Additionally, the rate of newly diagnosed diabetes in Mississippi residents was reported as 9.8% in 2018, 2.0% higher than the national average [1]. Diabetes was the seventh leading cause of death in Mississippi in 2019, with 38.9 per 100,000 deaths attributed to diabetes [2], compared to a national average of 26.7 deaths per 100,000 [3]. The economic burden of diabetes on Mississippi is substantial, as total direct and indirect costs of diagnosed diabetes mellitus in Mississippi were estimated at $3.4 billion in 2017 [4]. Considering the significant impact that diabetes has on Mississippians, it is vital to identify all factors that play a role in the high diabetes prevalence in Mississippi so physicians and public health officials can identify the most effective priorities for preventing and treating diabetes.

Adverse childhood experiences (ACEs) are potentially traumatic events such as childhood abuse (psychological, physical, or sexual) and stressful environmental circumstances such as household dysfunction (e.g., exposure to substance abuse, living with someone with mental illness, exposure to domestic violence, or living with someone who was incarcerated) that have been associated with the development of many chronic health conditions and significant causes of death in adulthood, including diabetes [5,6]. A recent systematic review and meta-analysis concluded that a significant association likely exists between ACEs and type 2 diabetes. They found that those who have experienced at least one ACE during childhood have a 32.0% increased risk of developing type 2 diabetes in adulthood [7]. Interestingly, unlike other diseases, many studies have found a threshold-response versus a dose-response relationship between ACEs and diabetes, suggesting that the disease can emerge later in life after reaching a threshold of cumulative stress [8]. Specifically, experiencing four or more categories of ACEs has been commonly associated with a significantly increased risk of developing diabetes [8].

In 2021, Mississippi had the ninth-highest prevalence (18.3%) of ACEs in the United States, with the national average being 14.8% [9]. Despite Mississippi's high prevalence of both ACEs and diabetes and the growing literature finding associations between ACEs and diabetes, little research has specifically examined a potential association among residents of Mississippi. To that end, the CDC's 2020 Behavioral Risk Factor Surveillance System (BRFSS) data [10] were utilized to investigate the relationship between ACEs and diabetes risk in Mississippi adults.

## Materials and methods

Data source

This study is a record-based study that reviewed previously recorded records. The data used in this study were from the 2020 Mississippi BRFSS survey, a randomized telephone survey administered by the CDC to non-institutionalized adults 18 years or older [10]. BRFSS collects information on health-related risk-taking behaviors, prevalence of chronic diseases, participation in preventive health services, and access to healthcare in the United States [10]. Data sampling using weights that account for nonresponse bias and non-coverage errors was used to generate representative estimates of the Mississippi population [11].

Data extraction and cleaning

Demographic information about study participants in Mississippi was obtained by selecting all respondents with state code 28 (Mississippi) from the 2020 BRFSS Core questionnaire data set. The demographic characteristics of respondents were obtained from the Core Section 8: Demographics section of the survey. Descriptive statistics, including frequencies and percentages, were calculated for categories of sex, age, race, level of education, income, and body mass index (BMI). Diabetes prevalence data were obtained from Module 2: Diabetes section of the survey from responses to the question asking "Has a doctor or other healthcare provider ever told you that you have diabetes?" Responses that reported "Yes, but female told only during pregnancy" and "No, pre-diabetes or borderline diabetes" were excluded from further analysis. ACE data were obtained from the 2020 Mississippi BRFSS survey's Module 21: Adverse Childhood Experiences, an 11-item module that asked individuals about their exposure to specific ACEs and exposure frequency. Questions that reported frequency categories of ACE exposure were collapsed into dichotomous variables representing the presence or absence of ACE exposure. Following the methodology outlined in a previous study [12], the 11 ACE survey questions were grouped into eight ACE exposure categories: Verbal Abuse, Physical Abuse, Sexual Abuse, Witnessed Domestic Violence, Household Substance Abuse, Household Member with Mental Illness, Parental Separation or Divorce, and Incarcerated Household Member. ACE categories encompassing multiple questions were assigned a value of 1/true for an individual if they reported a positive ACE exposure to at least one question within the category. Questions contained in the module were designated exposure categories as seen in Table 1.

**Table 1:** ACE exposure category by ACE exposure question prompt ACE: adverse childhood experience

ACE exposure question prompt	Combined exposure category
Did you live with anyone who was depressed, mentally ill, or suicidal?	Household Member with Mental Illness
Did you live with anyone who served time or was sentenced to serve time in a prison, jail, or other correctional facility?	Incarcerated Household Member
Were your parents separated or divorced?	Parental Separation or Divorce
How often did your parents or adults in your home ever slap, hit, kick, punch, or beat each other up?	Witnessed Domestic Violence
Not including spanking (before age 18), how often did a parent or adult in your home ever hit, beat, kick, or physically hurt you in any way?	Physical Abuse
How often did a parent or adult in your home ever swear at you, insult you, or put you down?	Verbal Abuse
How often did anyone at least five years older than you or an adult ever touch you sexually?	Sexual Abuse
How often did anyone at least five years older than you or an adult try to make you touch them sexually?	Sexual Abuse
How often did anyone at least five years older than you or an adult force you to have sex?	Sexual Abuse
Did you ever live with anyone who was a problem drinker or alcoholic?	Household Substance Abuse
Did you live with anyone who used illegal street drugs or who abused prescription medications?	Household Substance Abuse

Additionally, following methods outlined in studies utilizing BRFSS ACE data [6,12], an ACE exposure score was calculated for individual respondents by summating each positively reported ACE exposure category. An individual's ACE exposure score was calculated starting with a net-zero exposure and each positively reported exposure category adding one point. Frequencies and percentages were calculated for ACE exposure scores and categories.

Following these changes, data were weighted using the weighting variable _LLCPWT per BRFSS guidelines [11] to reflect geographic population estimates and reduce bias due to selection probability.

Binary logistic regression was used to determine factors that were independently associated with having ever been told by a physician that the respondent had diabetes. The variables analyzed included the ACE category, the number of ACE categories experienced, and demographic variables including sex, age, race, level of education, income, and BMI. Those variables determined to be associated with the outcome at a significance level of p≤0.10 were included in the multivariate model. Those variables that remained in the multivariate model at a significance level of p≤0.05 or lower were retained in the final model. The same approach was used to determine the odds of being diagnosed with diabetes across ACE exposure scores.

## Results

Table 2 shows sample demographics by diabetes status in Mississippi adults. The age group 65+ was the most frequently represented age category with 503,397 respondents (22.1%). Approximately 59% (1,323,791) were white, and 37% (844,344) were black. About 34% (776,743) of respondents had some college, and 40% (696,515) had an annual income of $50,000 or more. About 40% (846,822) of respondents were obese, 33% (706,538) were overweight, and 26% (543,373) were normal weight.

**Table 2:** Demographics by diabetes status in Mississippi adults n: number; %: percentage; +: plus; HS: high school; k: dollars in thousands; BMI: body mass index; <: less than; ≤: less than or equal to; ≥: greater than or equal to

Characteristics	Total sample n (%)	Yes diabetes n (%)	No diabetes n (%)
Population frequency	2,280,491 (100.0)	333,501 (14.6)	1,889,698 (82.9)
Sex			
Male	1,091,124 (47.8)	158,727 (47.6)	916,752 (48.5)
Female	1,189,367 (52.2)	174,774 (52.4)	972,946 (51.5)
Age			
18-24	252,889 (11.1)	5,997 (1.8)	243,740 (12.9)
25-34	431,886 (18.9)	21,150 (6.3)	398,110 (21.1)
35-44	363,114 (15.9)	25,458 (7.6)	329,557 (17.4)
45-54	355,491 (15.6)	57,621 (17.3)	291,124 (15.4)
55-64	373,714 (16.4)	85,351 (25.6)	279,386 (14.8)
65+	503,397 (22.1)	137,925 (41.4)	347,781 (18.4)
Race			
White	1,323,791 (58.6)	175,894 (53.0)	1,117,717 (59.7)
Black	844,344 (37.4)	142,812 (43.0)	676,325 (36.1)
Other	47,890 (2.1)	9,035 (2.7)	38,198 (2.0)
Multiracial	12,293 (0.5)	726 (0.2)	11,144 (0.6)
Hispanic	31,007 (1.4)	3,403 (1.0)	27,604 (1.5)
Education			
Less than HS	353,165 (15.5)	82,459 (24.8)	261,241 (13.9)
HS graduate	688,999 (30.3)	103,873 (31.2)	567,785 (30.1)
Some college	776,743 (34.2)	96,821 (29.1)	660,975 (35.1)
College graduate	453,738 (20.0)	49,442 (14.9)	393,643 (20.9)
Income			
Less than 15K	206,052 (11.9)	43,769 (17.9)	158,651 (11.0)
15k-25k	409,524 (23.6)	68,051 (27.8)	327,862 (22.6)
25k-35k	188,009 (10.8)	31,583 (12.9)	152,831 (10.6)
35k-50k	234,151 (13.5)	29,092 (11.9)	198,236 (13.7)
50k+	696,515 (40.2)	72,372 (29.6)	610,547 (42.2)
BMI			
Underweight (<18.5)	37,701 (1.8)	2,767 (0.9)	34,791 (2.0)
Normal weight (≤18.5-25)	543,373 (25.5)	38,936 (12.5)	496,809 (28.1)
Overweight (≤25-30)	706,538 (33.1)	88,051 (28.2)	605,850 (34.2)
Obese (≥30)	846,822 (39.7)	182,243 (58.4)	633,130 (35.8)

**Figure 1:**
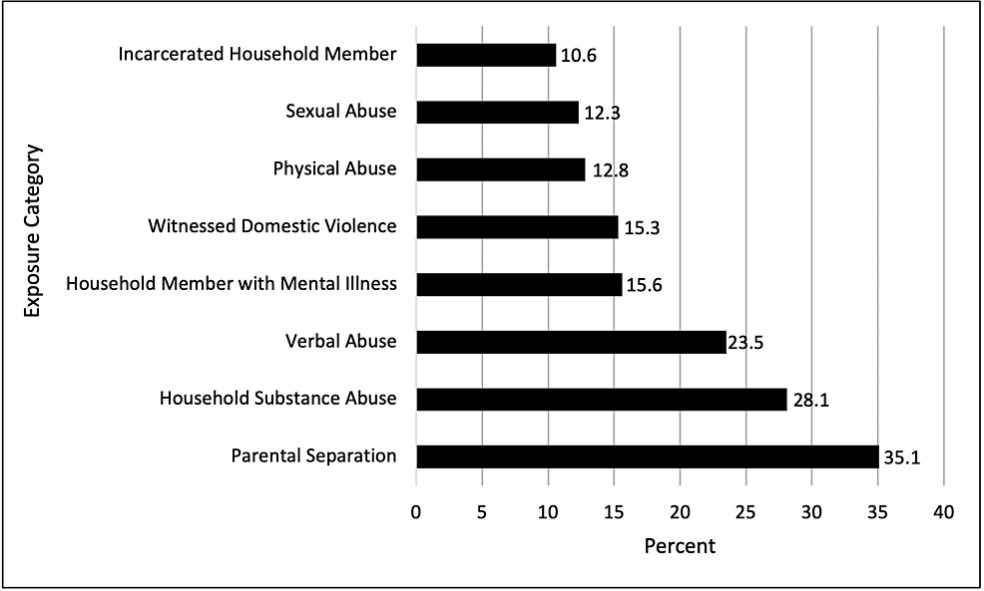
Prevalence of exposure to ACEs by category in Mississippi adults ACEs: adverse childhood experiences

The prevalence of each of the eight ACE categories among Mississippi adults was highly variable. Most notably, Parental Separation or Divorce was the most frequent ACE category experienced with 800,452 participants (35.1%) reporting they experienced it. Conversely, Incarcerated Household Member was the least frequently experienced ACE category among Mississippi adults with 241,732 participants (10.6%) reporting that experience (Figure 1). A statistically significant difference (p<0.001) was found in the prevalence of diabetes across all eight ACE categories. After adjusting for covariates, those who reported exposure to the Physical Abuse (AOR 1.72, 95% CI 1.69; 1.75) or Sexual Abuse (AOR 1.56, 95% CI 1.53; 1.58) categories had the highest odds of being diagnosed with diabetes. Additionally, respondents who reported exposure to the Household Member with Mental Illness, Parental Separation or Divorce, or Household Substance Abuse categories were more likely to have also reported a previous diabetes diagnosis on average (Table 3). In contrast, those who reported exposure to the Witnessed Domestic Violence, Incarcerated Household Member, or Verbal Abuse categories had decreased odds of reporting a previous diabetes diagnosis.

**Table 3:** Adjusted logistic regression models for the relationship between individual ACE categories and odds of diabetes *All categories were significant across diabetes status at p<0.001 **The model was adjusted for sex, age, race, level of education, income, and BMI ACE: adverse childhood experience; AOR: adjusted odds ratio; 95% CI: 95% confidence interval; BMI: body mass index

ACE exposure category*	AOR**	95% CI***
Physical Abuse	1.72	1.69-1.75
Sexual Abuse	1.56	1.53-1.58
Household Member with Mental Illness	1.36	1.34-1.38
Parental Separation	1.21	1.19-1.22
Household Substance Abuse	1.09	1.07-1.10
Verbal Abuse	0.92	0.90-0.93
Incarcerated Household Member	0.72	0.70-0.73
Witnessed Domestic Violence	0.70	0.69-0.71

**Figure 2:**
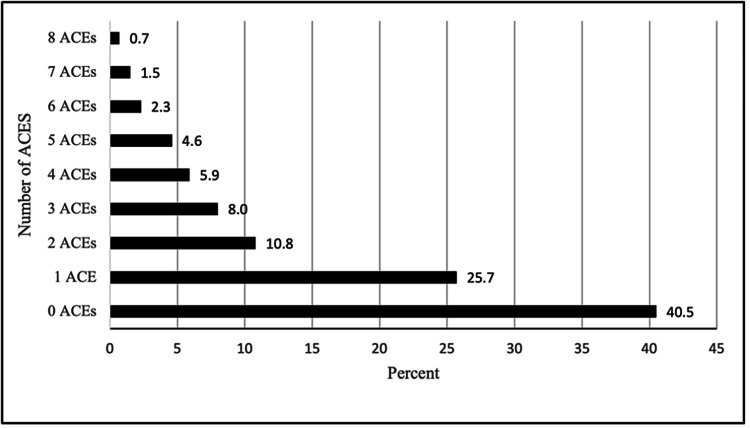
Distribution of ACE exposure scores among Mississippi adults ACEs: adverse childhood experiences

The distribution of ACE exposure scores can be found in Figure 2. The sum of ACEs experienced ranged from 0 to 8, with Mississippi adults reporting positive exposure to an average of 1.47 (±1.82) ACE categories. Among Mississippi respondents, 923,599 (40.5%) reported zero ACEs, 586,086 (25.7%) reported one ACE, and 246,293 (10.8%) reported two ACEs. Around 524,513 (23%) respondents experienced three or more ACEs. The adjusted odds ratio (AOR) of being diagnosed with diabetes for each ACE exposure score can be found in Table 4. All scores were significantly associated with ever being diagnosed with diabetes at p<0.001, except for one ACE (significant at p<0.01) and two ACEs (not significant). In general, as the ACE exposure score increased, the odds of being diagnosed with diabetes also increased. There were two exceptions. Having an ACE exposure score of 2 was not found to be significantly associated with ever being diagnosed with diabetes (AOR 0.94, 95% CI 0.92; 0.95). The odds of being diagnosed with diabetes for those with an ACE exposure score of 8 (AOR 1.66, 95% CI 1.57; 1.77) was lower than for those with an ACE exposure score of 7 (AOR 2.20, 95% CI 2.10; 2.31).

**Table 4:** Adjusted multivariate logistic regression model results for the relationship between ACE exposure scores and odds of diabetes *All ACE scores were significant at p<0.001, except for 1 ACE (significant at p<0.01) and 2 ACEs (not significant) **The model was adjusted for sex, age, race, level of education, income, and BMI ACEs: adverse childhood experiences; AOR: adjusted odds ratio; 95% CI: 95% confidence interval; BMI: body mass index

ACE exposure score*	AOR**	95% CI***
1 ACE	1.02	1.01-1.03
2 ACEs	0.94	0.92-0.95
3 ACEs	1.39	1.37-1.42
4 ACEs	1.34	1.31-1.38
5 ACEs	1.55	1.51-1.59
6 ACEs	2.08	2.02-2.15
7 ACEs	2.20	2.10-2.31
8 ACEs	1.66	1.57-1.77

## Discussion

In order to determine if the relationship between ACE exposure scores and diabetes diagnosis followed a dose-dependent or threshold response, ACE exposure scores were treated as categorical variables for analysis purposes. Many of the ACE categories, including Physical Abuse, Sexual Abuse, Household Member with Mental Illness, Parental Separation or Divorce, and Household Substance Abuse, were each independently associated with an increased likelihood of being diagnosed with diabetes in this study. In contrast, some of the ACE categories, including Verbal Abuse, Incarcerated Household Member, and Witnessed Domestic Violence, each showed a decreased likelihood of being diagnosed with diabetes. However, this variance in risk across ACE exposure categories is not unique to this study. Similar to a previous study [13], this study found that the odds of developing diabetes were the highest for those who experienced ACEs related to sexual and physical abuse, while having an incarcerated parent seemed to have a protective effect.

The current study found that the more ACE categories an individual experiences, the more likely they were to have ever been diagnosed with diabetes, unless the score was 2 or 8. However, we did not see a threshold response that has been reported in a previous study on the subject [8]. The absence of a threshold response in this study's results may be due to the unique characteristics of the Mississippi population, where other risk factors for diabetes development other than ACEs might already make Mississippi adults more prone to developing diabetes, making exposure to multiple ACEs unnecessary to reach a threshold of increased risk. For example, Mississippi adults' lack of physical exercise [14] may predispose them to diabetes development independent of ACE exposure.

Models of ACE modification of risk of diabetes development

The link between ACEs and diabetes risk is multifactorial, and much research in recent years has begun to suggest the potential mechanisms behind the phenomenon being discussed here; for example, a previous study [15] on ACEs and chronic health conditions has hypothesized that risky health behaviors (RHBs) (e.g., high BMI, smoking, alcohol abuse) may be a crucial mediator between the positive association between ACE exposure and the development of chronic health conditions, including diabetes. One study examining this relationship [15] found that, although many RHBs were associated with exposure to ACEs, BMI might be the most important mediator between ACEs and diabetes. The underlying mechanism responsible for the relationship between ACEs and obesity may involve attempts by those exposed to chronic psychological stress or trauma to regulate their emotions by overeating [16].

Another critical mediator between ACEs and the development of diabetes involves the effect of ACEs on access to healthcare and the use of preventive healthcare services. One study found that ACEs were inversely associated with having health insurance, having a primary care provider, and receiving regular checkups, even after controlling for other important sociodemographic factors [17].

Other researchers have examined ACEs' molecular and epigenetic effects and their impact on diabetes and other chronic health conditions. A previous study has shown that chronic stress during childhood can negatively affect the hypothalamic-pituitary-adrenal (HPA) axis and over-activate the sympathetic nervous system through overall increases in the secretion of cortisol and catecholamines, resulting in emotion dysregulation, insulin resistance, central obesity, and metabolic syndrome [18]. Additionally, one study examining the effects of childhood adversity on deoxyribonucleic acid (DNA) methylation profiles concluded that childhood abuse resulting in genome-wide methylation profiles could significantly impact long-term health into adulthood [19]. Lastly, a recent systematic review [20] concluded that there was a significant association between childhood maltreatment and increased levels of inflammatory cytokines. Many of these cytokines have been found to play a role in the development of type 2 diabetes [21].

The prevention of ACEs is an important goal for the CDC. They have recommended six strategies [5] including strengthening economic support to families (e.g., strengthening household financial security, family-friendly work policies), promoting social norms that protect against violence and adversity (e.g., public education campaigns, legislative approaches to reduce corporal punishment), ensuring a solid start for children (e.g., high-quality child care, pre-school enrichment with family engagement), teaching skills to children and adults (e.g., social-emotional learning, parenting skills, and family-interpersonal relationship approaches), connecting youth to caring adults and activities (e.g., mentoring programs, after-school programs), and intervening to lessen immediate- and long-term harms (e.g., enhanced primary care, treatment to reduce the damages of ACEs). It may prove vital for Mississippi legislators and physicians to implement these suggested strategies to prevent and lessen the impact of ACEs and their effect on increased diabetes risk in Mississippians.

Strengths and limitations

A notable strength of this study is its large sample size and breadth of survey data. Because there are many confounding socioeconomic factors unique to Mississippi, the data used in this study have the potential to reveal patterns of association between ACEs and diabetes risk that may be masked by studies using data from many different states. The survey sampling and analysis methods employed in this study allow for greater precision in analysis by accounting for specific characteristics within the state and by limiting exposure to potential confounding variables introduced by sampling a population with a larger geographic range. This consideration is important given the magnitude of the impact that diabetes has had on the socioeconomic climate of Mississippi, which is extreme in comparison to many other states in the country.

This study had several key limitations that should be considered when interpreting its findings. The first of these limitations was that survey data was collected in a manner that did not distinguish between type 1 and type 2 diabetes. In the BRFSS survey, responses are recorded to include positive and negative responses when asking about previous diabetes diagnosis, but the 2020 BRFSS survey did not contain sub-modules to distinguish between type 1 and type 2 diabetes. However, due to the high proportional prevalence of type 2 (91%) over type 1 (6%) diabetes [22], the results are assumed to largely reflect changes in odds associated with type 2 diabetes diagnosis for this study.

Additionally, there are many temporal aspects of ACEs that we could not assess in this study such as severity, age of onset, and cumulative exposure that may have a significant association with the risk of a diabetes diagnosis. This is a limitation intrinsic to cross-sectional study designs. Future longitudinal studies should be conducted to gather more information on the potential mechanisms whereby ACEs modify the risk of diabetes development.

Additional sources of error in the study can be attributed to the self-reporting nature of survey data collection. This is particularly concerning for ACE reporting as the nature of the questions can be understandably sensitive. Given that ACEs were a key variable in this study, it is expected that some degree of error can be attributed to over- and under-reporting of certain categories of ACE exposure. Additionally, other self-reported data such as BMI and actual diabetes status may be subject to reporting bias.

Lastly, while this study controlled for confounding demographic variables such as income, race, level of education, and BMI, many other variables can confound the analysis of the relationship between diabetes and ACEs. This study aimed to control the variables known to significantly influence the risk of developing diabetes. However, there is ample opportunity to expand the scope of this model to address many additional confounding variables.

## Conclusions

Overall, this study shows a strong association between ACEs and the development of diabetes in Mississippi adults. While more research is needed to explain the relationship between ACEs and diabetes risk fully, this relationship likely represents an important area for prevention efforts in legislative and public health campaigns, as well as universal screening procedures in primary care that can decrease the prevalence and burden of diabetes in the state of Mississippi.
